# Urinary Liver-Type Fatty Acid Binding Protein, a Biomarker for Disease Progression, Dialysis and Overall Mortality in Chronic Kidney Disease

**DOI:** 10.3390/jpm13101481

**Published:** 2023-10-11

**Authors:** Nicos Mitsides, Vikram Mitra, Ananya Saha, Shelly Harris, Philip A. Kalra, Sandip Mitra

**Affiliations:** 1 Medical School, University of Cyprus, 2029 Nicosia, Cyprus; 2 Department of Nephrology, Nicosia General Hospital, 2029 Nicosia, Cyprus; 3 John Radcliffe Hospital, Oxford University Medical School, Headington, University of Oxford, Oxford OX1 2JD, UK; vikram.mitra5@gmail.com; 4 Manchester Institute of Nephrology and Transplantation, Department of Research and Innovation, Manchester University Hospitals, Manchester M13 9WL, UK; ananya.saha@mft.nhs.uk (A.S.); shelly.harris@mft.nhs.uk (S.H.); 5 Department of Renal Medicine, Salford Royal Hospital, Northern Care Alliance NHS Foundation Trust, Manchester Academic Health Sciences Centre, Salford M6 8HD, UK; philip.kalra@nca.nhs.uk; 6 Manchester Institute of Nephrology and Transplantation, Manchester University Hospitals & University of Manchester, Manchester Academic Health Sciences Centre, Manchester M13 9WL, UK

**Keywords:** chronic kidney disease, biomarkers, urinary liver-type fatty acid binding protein, urinary protein-to-creatinine ratio

## Abstract

Chronic kidney disease (CKD) is a major public health concern with an increasing proportion of sufferers progressing to renal replacement therapy (RRT). Early identification of those at risk of disease progression could be key in improving outcomes. We hypothesise that urinary liver-type fatty acid binding protein (uL-FABP) may be a suitable biomarker for CKD progression and can add value to currently established biomarkers such as the urinary protein-to-creatinine ratio (uPCR). A total of 583 participants with CKD 1–5 (not receiving renal replacement therapy) entered a 2 yr prospective longitudinal study. UPCR and uL-FABP were measured at baseline and CKD progression was defined as either (i) a decline in eGFR of >5 mL/min/1.73 m^2^ or an increase in serum creatinine by 10% at 1 yr; (ii) a decline in eGFR of >6 mL/min/1.73 m^2^ or an increase in serum creatinine by 20% at 2 yrs; or (iii) the initiation of RRT. A combined outcome of initiating RRT or death was also included. Approximately 40% of participants showed CKD progression. uL-FABP predicted CKD progression at both years 1 and 2 (OR 1.01, *p* < 0.01). Sensitivity and specificity were comparable to those of uPCR (AUC 0.623 v 0.706) and heat map analysis suggested that uL-FABP in the absence of significant proteinuria can predict an increase in serum creatinine of 10% at 1 yr and 20% at 2 yrs. The risk of the combined outcome of initiating RRT or death was 23% higher in those with high uL-FABP (*p* < 0.01) independent of uPCR. uL-FABP appears to be a highly sensitive and specific biomarker of CKD progression. The use of this biomarker could enhance the risk stratification of CKD and its progression and should be assessed further.

## 1. Introduction

Affecting approximately 10–13% of the population worldwide [1], the epidemic of chronic kidney disease (CKD) has soared through the ranking of causes of global mortality, rising from 27th place in 1999 to 18th in 2010 [2,3] to 12th in 2017 [4]. Established as a major public health concern [5], CKD is diagnosed, classified and monitored based on estimates of renal function and creatinine clearance. Using the Kidney Outcomes Quality Initiative (KDOQI) classification [6] CKD can be defined in stages (1–5) according to disease severity, based on estimates of the glomerular filtration rate (eGFR) derived using either the Modification of Diet in Renal Disease Study Group (MDRD) [7] methodology or more recently the Chronic Kidney Disease Epidemiology Collaboration (CKD-EPI) [8] equations and the presence or absence of proteinuria. The risk of disease progression increases at advanced CKD stages with 8.9% of CKD stage 4 patients and 39.1% of CKD stage 5 patients likely to show a decline in eGFR of 5 mL/min/1.73 m^2^/year or more [9]. However, only 0.5% of the general population progress to these advanced stages of CKD; additionally, the majority of patients with CKD have stage 3 disease (7–9% of the general population; 80–90% of all CKD) and will never progress to end-stage disease [10]. Nevertheless, 6% of CKD stage 3 patients show a decline in eGFR of 5 mL/min/1.73 m^2^/year [11]. Globally, 50–400 per million CKD patients will become dependent on renal replacement therapy per year [12], alongside an increased risk of cardiovascular disease [13,14]. In this context, early identification of patients at risk of CKD progression is imperative.

Although eGFR is a useful measure for stratifying disease severity, it provides little information about the potential risk of disease progression [15]. Proteinuria has been an important prognostic marker in glomerular pathologies [16], especially diabetic nephropathy [17], but is limited as a biomarker for CKD progression and response to treatment [18]. A number of alternative biomarkers or risk prediction equations based on creatinine and uPCR (Kidney Failure Risk Equation) have been proposed as predictors of disease progression [15]. Urinary liver-type fatty acid binding protein (uL-FABP) is one such biomarker with the potential for predicting CKD progression. Free fatty acids are found in abundance in renal tubules in proteinuric renal disease and they have been implicated in the pathogenesis of proximal tubular injury through their binding to albumin and activation of the inflammatory response [18]. This phenomenon is also present ischemia and under oxidative stress [19,20], in the absence of proteinuria. Free fatty acids are believed to bind to the 14 kDa fatty acid binding protein 1 (also known as L-FABP), found in the cytoplasm of renal tubular cells, before being transported to mitochondria or peroxisomes to be metabolized by oxidation [19,20,21]. uL-FABP is already established as a reliable biomarker of acute kidney injury [21,22] and, in a number of small studies, has been linked with CKD progression in diabetic [23,24,25] and non-diabetic [26] patients.

Our aim was to assess whether uL-FABP levels can predict a decline in renal function, and thus disease progression, in patients with CKD attending outpatient clinics in two large tertiary centres in the United Kingdom.

## 2. Materials and Methods

This was a 2 yr prospective follow-up study of participants with CKD stages 1–5 (not receiving renal replacement therapy) attending outpatient nephrology clinics in 2 large tertiary university renal centres in the northwest of the UK. The study received approval from the Yorkshire and Humber-Leeds West NHS Research Ethics Committee (15/YH/0516) and was adopted on the NIHR clinical research network portfolio (CRN ID: 20484). Adult patients, with capacity to consent, receiving their care in the participating centres were eligible to participate in the study and were approached during their attendance to clinic. Participation in the study was voluntary. Informed written consent was obtained prior to enrolment. 

### 2.1. Definitions

A number of definitions of CKD progression were considered; however, based on the definitions used by NICE [2,3] and KDIGO [6], for purposes of this study at the time, CKD progression was defined as either: 

(A) at 1 yr follow up, (i) a decline in eGFR by the equivalent of 5 mL/min/1.73 m^2^, (ii) increase in serum creatinine by 10% or (iii) initiation of renal replacement therapy for CKD 5; 

(B) at 2 yr follow up, (i) a decline in eGFR by the equivalent of 6 mL/min/1.73 m^2^, (ii) increase in serum creatinine by 20% or (iii) initiation of renal replacement therapy. 

The combined outcome of initiating renal replacement therapy or death was also evaluated.

### 2.2. Study Population Size

Katoh et al. [27] suggest that a cut-off of 19 μg/g.cr for urinary L-FABP carries 100% sensitivity and 81.8% specificity for the detection of contrast-induced AKI. Their mean study population uL-FABP level was 59.8 ± 45.6 μg/g.cr. Shingai et al. [28] used a detection cut-off 8.4 μg/g.cr based on the study of 420 healthy volunteers by Kamijo-Ikemori et al. [24]. Although published data from Kamijo et al. [26] suggest that a uL-FABP level of 17.4 μg/g.cr can be used as a cut-off for monitoring progression in CKD, the use of uL-FABP as a predictor of progression of CKD is unclear and remains under investigation. Although the literature failed to inform any power calculation, we believe that based on previous study population sizes, a population of 500 CKD participants would provide us with an adequate sample size to evaluate the potential of uL-FABP as a CKD biomarker.

### 2.3. Data Collection

The past medical history and medication lists were collected directly from participants and through their electronic medical records. These were used to calculate the Davies’ Comorbidity Score for all participants [29] as an indicator of coexisting disease burden. Prospective follow-up data on CKD were collected from patient electronic records at 1 yr and 2 yrs from enrolment.

### 2.4. Blood Sampling, eGFR Calculation and CKD Classification

Serum samples were collected as part of participants’ routine attendance to clinic and were analysed for urea and creatinine at the individual participating centres’ NHS biochemistry laboratories. Both laboratories comply with the NHS laboratory standardization process for clinical laboratories. The eGFR was calculated using the MDRD [7] formula. Patients were categorised in CKD stages according to KDOQI [30]. The difference in creatinine and eGFR over the 1st and 2nd years of the study was taken to be the difference in the values of these parameters at the data collection time points and at baseline.

### 2.5. Urine Sampling and Analysis

A 20 mL urine sample was provided as part of the participants’ routine clinical care. Then, 10 mL was analysed by the participating centres’ local NHS biochemistry laboratories to measure urinary protein, creatinine and the protein: creatinine ratio (uPCR). The remaining 10 mL sample was stored at −80° pending measurement of uL-FABP levels using an Enzyme-Linked Immunosorbent Assay (ELISA).

### 2.6. ELISA Analysis for the Measurement of uL-FABP Levels

Urine samples were processed in batches. Samples were thawed overnight and centrifuged at 2400 rpm at room temperature for 10 min to separate and remove impurities. uL-FABP levels were quantified using a commercially available ELISA kit (CMIC Co Ltd., Tokyo, Japan) as per the manufacturers’ instructions. All samples were assessed in duplicate within the same ELISA plate. The intra-assay coefficient of variation was 5.5%. Ten samples with detectable levels of uL-FABP were analysed separately to assess inter-plate assay variability. The inter-assay coefficient of variance was 7.8%. Calculations of uL-FABP levels were adjusted for urinary creatinine concentration obtained from the uPCR data that were measured within the same sample. 

### 2.7. Statistical Analysis

The demographic and biochemical characteristics of participants were analysed using descriptive epidemiology. Categorical variables were presented as frequencies and percentages. Continuous variables with normal distribution were presented as means and standard deviation (SD) while variables with skewed distribution were presented as medians with minimum and maximum values. Normality of distribution was assessed using the Shapiro–Wilk method. The relationship between baseline urine uL-FABP levels, serum creatinine, eGFR and urine PCR was explored using stepwise linear regression modelling adjusted for age and sex. Skewed outcome variables underwent logarithmic transformation. Longitudinal analysis was performed from the change in eGFR, increase in serum creatinine, CKD progression and combined renal mortality. Participants that received kidney transplantation between baseline and 1 yr were removed from year one analysis unless they had initiated dialysis prior to transplantation. The same was true for participants receiving renal transplantation between years 1 and 2 for year 2 analysis. The association between uL-FABP and uPCR and the change in serum creatinine and eGFR was assessed using stepwise linear regression modelling adjusted for age and sex. The demographic characteristics and biochemical profiles of participants that exhibited CKD progression were compared to those without disease progression using group comparison. Categorical variables were compared using Pearson’s Chi^2^ with observation of the standardised residuals as post hoc analysis. Continuous variables were skewed in distribution, so differences between the assessed groups were analysed using the Kruskal–Wallis test. The predictive potential of uL-FABP was evaluated using logistic regression modelling adjusted for age, sex and proteinuria. The predictive sensitivity and specificity of uL-FABP were evaluated through ROC curve analysis and were compared to that of uPCR and uPCR in combination with uL-FABP. The interaction between uL-FABP and uPCR and changes in creatinine was also explored using heat map assessment. Subgroup analysis stratified by individuals’ CKD stage at baseline and the absence of proteinuria was performed using the same logistic regression model utilised for the entire study population. Combined outcome analysis was performed using Cox regression modelling using 8 mcg/gCr of uL-FABP as a cut-off for a significant level. Models were adjusted for age, sex and uPCR. Statistical analyses were performed by IBM SPSS Statistics, version 23 (IBM Corp., Armonk, NY, USA).

## 3. Results

**Figure 1:**
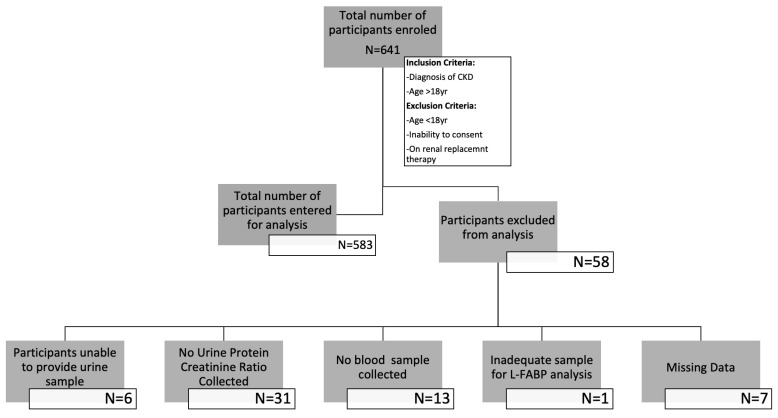
Flow diagram illustrating the number of participants enrolled in the study and those whose data were entered for analysis.

**Figure 2:**
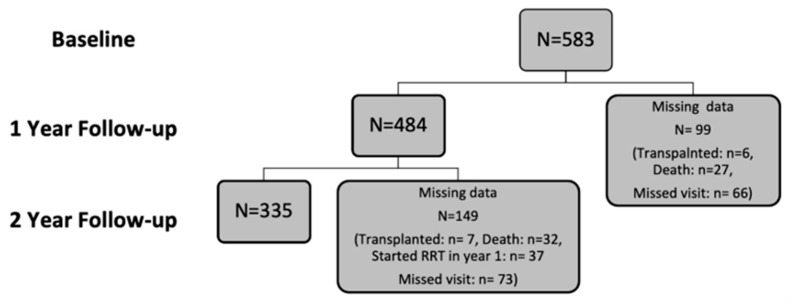
Flow diagram illustrating the number of participants analysed per year of study and study dropout.

**Figure 3:**
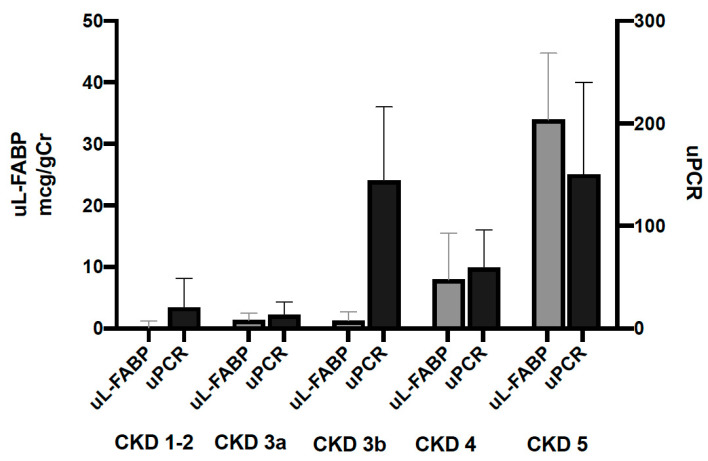
Urinary levels of L-FABP and PCR at baseline stratified by CKD stage. Bars represent median biomarker levels and whiskers indicate the 95% confidence interval.

A total of 641 patients were recruited into the study. Of those recruited, the data of 583 participants were analysed at baseline, while 484 were available in year one and 335 in year two. Participants’ exclusions and dropout reasons are shown in Figure 1 and Figure 2. The demographic characteristics and biochemical profiles of these participants are shown in Table 1 and Figure 3. No participants were noted to have liver disease.

At baseline, uL-FABP levels correlated to those of uPCR (beta = 0.383, CI 0.305; 0.457, *p* ≤ 0.001, R^2^ = 0.142). uL-FABP also correlated to serum creatinine (beta = 0.379, CI 0.303; 0.455, *p* ≤ 0.001, R^2^ = 0.145) and eGFR (beta = −0.380, CI −0.454; −0.302, *p* ≤ 0.001, R^2^ = 0.143). This association was independent of proteinuria, age and sex (linear regression models shown in Table 2).

### 3.1. Year 1 and 2 Analysis 

The median percentage increase in serum creatinine between baseline and year 1 was 3.5% (−6.9; 18.8) with a median decrease in MDRD eGFR of 1 mL/min (−2.0; 5.0).

Both uL-FABP (beta = 0.140, CI 0.045; 0.203, *p* = 0.002, R^2^ = 0.020) and uPCR (beta = 0.202, CI 0.102; 0.258, *p* < 0.001, R^2^ = 0.041) correlated with increases in serum creatinine. UPCR (beta = 0.101, CI 0.010; 0.159, *p* = 0.026, R^2^ = 0.010) predicted a decrease in eGFR over 1 year, but this relationship was not observed with uL-FABP (beta = −0.045, CI −0.112; 0.037, *p* = 0.324, R^2^ = 0.002).

By the end of year 1, 208 participants (43%) showed progression of CKD defined as a reduction in eGFR by 5 mL/min, an increase in serum creatinine by 10% or initiation of renal replacement therapy. Their characteristics are described in Table 3. Both uL-FABP and uPCR predicted CKD progression (logistic regression model adjusted for sex and age) (Table 4). 

By the end of year 2, 153 participants (42%) showed CKD progression defined as a reduction in eGFR by 6 mL/min, an increase in creatinine by 20% and start of renal replacement therapy. uL-FABP did not correlate with the increase in creatinine over 2 years (beta = 0.041, *p* = 0.464) or the decrease in eGFR (beta = −0.030, *p* = 0.602) over the same period. uPCR did correlate with both of these outcomes in a linear model adjusted for age, sex and uL-FABP (ΔCreatinine: beta = 0.310, *p* < 0.001, ΔeGFR; beta = 0.253, *p* < 0.001). Both L-FABP and uPCR predicted CKD progression (logistic regression model adjusted for sex and age). The results are shown in Supplementary Table S1.

### 3.2. Sensitivity and Specificity

**Figure 4:**
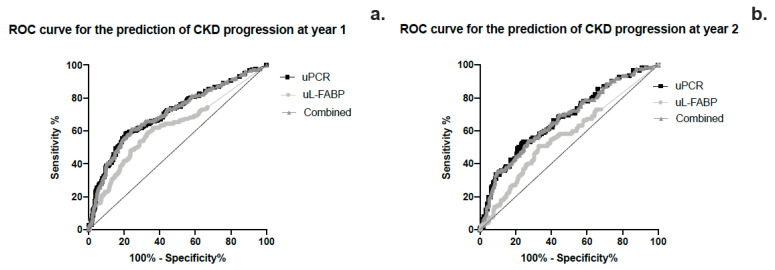
(**a**,**b**) ROC curve analysis of the sensitivity and specificity of urinary L-FABP, PCR and the combined effect of the 2 biomarkers in predicting CKD progression at years 1 and 2.

**Figure 5:**
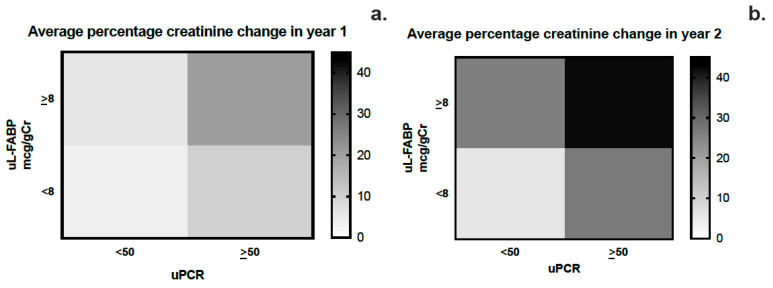
(**a**,**b**) Heat map analysis of the average change in creatinine at years 1 and 2 stratified by urinary L-FABP and PCR levels.

The area under the curve for sensitivity and specificity of uL-FABP in predicting CKD progression was calculated using ROC curve analysis as 0.623 (CI 0.572; 0.675, *p* < 0.01) while that for uPCR was 0.706 (CI 0.658; 0.753, *p* < 0.01) (Figure 4a,b). Heat map analysis (Figure 5) also showed that uL-FABP in the absence of significant proteinuria can predict modest increases in serum creatinine (10% at 1 year and 20% at 2 years). The combined elevation of uL-FABP and uPCR had the highest predictive power for increases in creatinine at both 1 year (≤20% increase; Figure 5a) and 2 years (≤40% increase; Figure 5b). 

### 3.3. Mortality and RRT Initiation Risk Analysis

**Figure 6:**
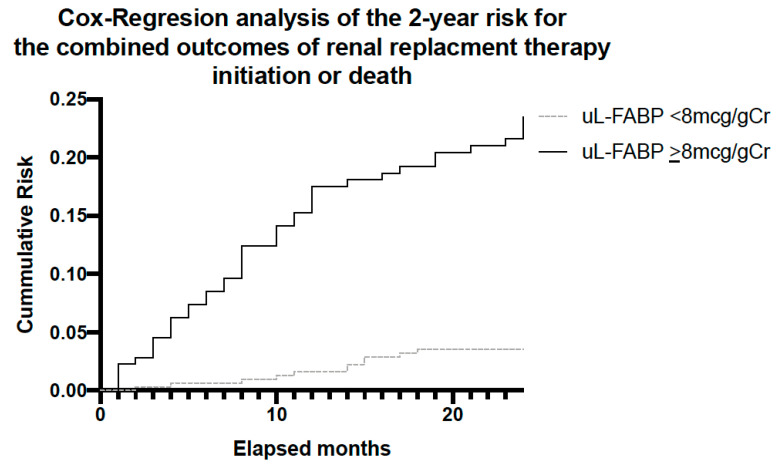
Analysis for the combined outcome of RRT initiation or death over the 2-year follow-up period stratified by participants uL-FABP levels at baseline.

Cox regression for the combined outcomes of death and initiation of RRT over the 2-year study period showed that the cumulative risk for patients with high uL-FABP levels (using uL-FABP of 8 as the differentiator) was significantly higher than those with low levels (*p* < 0.001) in a model adjusted for age, sex and uPCR (Figure 6).

## 4. Discussion

We describe the findings of a prospective 2-year follow-up study exploring the potential of uL-FABP as a biomarker for disease progression in CKD. This is the largest study of a CKD population using uL-FABP. Our baseline analysis demonstrated an association between uL-FABP levels and disease severity, as demonstrated both by raised creatinine and reduced eGFR but also proteinuria. The presence of elevated levels of uL-FABP at baseline appears to predict CKD progression both at 1 and 2 years of follow-up. Although proteinuria appears to have a higher sensitivity and specificity than uL-FABP alone in predicting CKD progression, our ROC curve analysis would suggest that the best prediction for sensitivity and specificity is offered by the combination of the two biomarkers. As apparent from the heat map analysis, patients with elevated uL-FABP levels were more likely to experience a rise in serum creatinine both at 1 and 2 years of follow-up. In addition to disease progression, elevated uL-FABP carried a 24% higher risk of RRT and overall mortality independent of the presence of proteinuria.

Serum creatinine levels, and by extension creatinine-based estimations of GFR, might portray a reliable insight into CKD severity but their overall value in predicting disease progression is limited [15]. uPCR and its risk prediction equations (KFRE [31]) remain the gold standard in identifying individuals more likely to progress [18,32,33,34] but our findings suggest that the absence of proteinuria does not exclude progressive pathology. In these individuals, the presence of elevated uL-FABP might be a better predictor of disease progression.

Urinary PCR and L-FABP biomarkers are likely to represent different pathological processes involving the kidney. While uPCR tends to be predominantly glomerular in its origin in the early stages of CKD [16], uL-FABP is produced by the renal tubules in response to ischemia and oxidative stress. A number of studies have linked urinary levels of L-FABP with tubular injury both in the acute and chronic phases of kidney disease. In health, L-FABP is produced in the proximal renal tubule and binds fatty acids reabsorbed by the renal tubular cells enabling their transposition into the tubular mitochondria [20]. Under ischaemic conditions, this process is compromised by lipid peroxidation products binding to L-FABP instead, leading to increased secretion of L-FABP into the urine [20]. Whether this process is pathological or protective is unclear. All studies to date have shown that high levels of uL-FABP are generally linked with adverse outcomes. This has been predominantly in the setting of acute kidney injury [35,36,37,38,39], perioperative ischaemic [40,41], contrast-induced acute tubular injury [42] and peri-haematopoietic stem cell transplantation-associated acute kidney injury [28]. In the setting of CKD, studies have been few, with limited study populations [23,26,38,43]. Some studies have investigated specific CKD groups such as those with diabetes or cardiovascular disease [23,26,38]. They do all, however, conclude that uL-FABP is a useful biomarker in CKD [20] and its potential should be explored further. Our results are suggestive that in addition to an additive predictive value to that of uPCR, uL-FABP might play a role in risk stratification of patients, especially with negligible or absent proteinuria. Our attempts to characterise this group were limited by a smaller subgroup size (Supplementary Table S2).

Although the association between uL-FABP and ESKD/RRT has been noted before in a study of diabetic Pima Indians [38], this is the first time that such an association has been shown in a large population of unselected patients with CKD.

Another potential benefit when considering uL-FABP as a potential biomarker of CKD progression in the clinical setting is its potential availability as a semi-quantitative point-of-care test. The technology can be adapted for screening the CKD population in clinics.

The main limitation of our study was the lack of repeated measurements of uL-FABP during the follow-up period. The limited number of studies in the field made the design of this study and any population size estimation difficult. Efforts were made to minimise selection biases during the recruitment of participants. Participants were randomly approached for recruitment during their attendance at routine CKD clinical appointments. Laboratory analysis was also blinded to the personal information of participants. 

In summary, uL-FABP appears to be a highly sensitive and specific biomarker of renal dysfunction and an effective predictor of CKD progression. A rapid assay of elevated uL-FABP in routine CKD care could improve rapid diagnosis and feasibility in clinical practice, especially in CKD patients with minimal or no proteinuria. 

Further studies involving large registries are needed to evaluate the role of uL-FABP in the risk stratification of CKD patients and disease progression. Future research should also investigate interventions that can modify the expression of the biomarker and help improve the prognosis of chronic kidney disease. 

## Figures and Tables

**Table 1:** Demographic and biochemical characteristics at baseline of participants entered for baseline and longitudinal analysis.

Demographics		Baseline	1 Year	2 Year
N		583	484	335
CKD stage	1–2	14.6%	13.6%	7.8%
	3A	13.3%	12.8%	15.6%
	3B	24.8%	24.8%	29.0%
	4	32.8%	33.5%	38.3%
	5	14.6%	15.3%	9.3%
Age (yrs)		65 (51; 75)	65 (51; 74)	65 (52; 74)
Sex: Male		56%	55.8%	54.0%
Ethnicity	White	78.3%	78.3%	79.4%
	Black	3.8%	3.7%	3.6%
	Asian	8.5%	9.1%	9.0%
	Chinese	1.0%	1.2%	1.2%
	Other	6.6%	6.0%	5.7%
	Unspecified	1.7%	1.7%	1.2%
Primary CKD Pathology	ADPKD	8.1%	8.5%	9.0%
	Diabetic Nephropathy	10.7%	10.7%	9.6%
	Glomerulonepgritis	16.5%	17.6%	17.3%
	Acute/Chronic TIN	3.3%	3.7%	4.8%
	Obstructive/Stones/Reflux	10.7%	10.7%	10.4%
	Renovascular/HTN/Ischaemic	12.8%	12.4%	11.3%
	Vasculitis/SLE	9.9%	11.0%	13.4%
	Myeloma	1.4%	1.4%	1.2%
	Hereditary Nephropathy	0.7%	0.8%	0.6%
	Other	9.4%	8.3%	9.0%
	Uncertain Aetiology	16.5%	14.9%	13.4%
Cardiovascular Disease		21.5%	20.2%	18.8%
Diabetes Mellitus		29.1%	28.5%	26.9%
Davies’ Comorbidity Score		1 (1; 2)	1 (1; 2)	1 (1; 2)
Number of Medications		7 (4; 9)	7 (4; 9)	7 (4; 9)
Number of Blood Pressure Medications		2 (1; 3)	2 (1; 3)	2 (1; 3)
ACEi/ARB	ACEi	33.4%	34.1%	34.4%
	ARB	21.5%	22.3%	25.4%
	Both	2.4%	2.5%	2.4%
Aldosterone Inhibitors		2.6%	2.1%	2.4%
Serum Creatinine (mmol/L)		173 (199; 265)	174 (124; 268)	169 (126; 238)
MDRD eGFR (mL/min/1.73 m^2^)		31 (19; 46)	30 (18; 46)	31 (21; 46)
uPCR mg/gCr		40 (11; 147)	42 (12; 152)	36 (11; 128)
uL-FABP ELISA (mcg/gCr)		2.79 (0; 19.4)	3.49 (0; 19.6)	2.68 (0; 16.4)

ACEi = angiotensin-converting enzyme inhibitors, CKD = chronic kidney disease, eGFR = estimate glomerular filtration rate, ELISA = enzyme-linked immunosorbent assay, HTN = hypertensive nephropathy, L = litre, m = metre, mcg = microgram, MDRD = Modification of Diet in Renal Disease, mg = milligram, mL = millilitre, mmol = millimol, SLE = systemic lupus erythematosus, TIN = tubulointerstitial nephritis, uL-FABP = urinary liver-type fatty acid binding protein, uPCR = urinary protein-to-creatinine ratio.

**Table 2:** Unadjusted and adjusted linear regression models of uL-FABP and uPCR with serum creatinine and eGFR.

Outcome: MDRD eGFR (Log^10^ eGFR)						
	Unadjusted (R^2^ = 0.169)		+Age (R^2^ = 0.313)		+Age, Sex (R^2^ = 0.317)	
	Beta (95% CI)	Sig	Beta (95% CI)	Sig	Beta (95% CI)	Sig
uPCR	−0.178 (−0.257; −0.096)	<0.001 *	−0.216 (−0.228; −0.140)	<0.001 *	−0.216 (−0.289; −0.141)	<0.001 *
uL-FABP	−0.313 (−0.392; −0.231)	<0.001 *	−0.300 (−0.372; −0.225)	<0.001 *	−0.300 (−0.372; −0.225)	<0.001 *
Outcome: Serum Creatinine (Log^10^ serum creatinine)						
	Unadjusted (R^2^ = 0.173)		+Age (R^2^ = 0.268)		+Age, Sex (R^2^ = 0.312)	
	Beta (95% CI)	Sig	Beta (95% CI)	Sig	Beta (95% CI)	Sig
uPCR	0.185 (0.104; 0.265)	<0.001 *	0.216 (0.139; 0.291)	<0.001 *	0.211 (0.135; 0.284)	<0.001 *
uL-FABP	0.312 (0.230; 0.392)	<0.001 *	0.302 (0.225; 0.377)	<0.001 *	0.307 (0.231; 0.379)	<0.001 *

Outcome variables with skewed distribution of errors underwent logarithmic transformation. Results are presented as beta coefficients with 95% confidence intervals (CIs). * denotes statistical significance (sig) at the level of *p* < 0.05. eGFR = estimate glomerular filtration rate, MDRD = Modification of Diet in Renal Disease, uL-FABP = urinary liver-type fatty acid binding protein, uPCR = urinary protein-to-creatinine ratio.

**Table 3:** Participants’ demographic characteristics and biochemical profiles stratified by CKD progression by the end of year 1 of follow-up.

Demographics		CKD Progression	No CKD Progression	*p*-Value
N		208	276	
CKD stage	1–2	8.5%	11.3%	<0.001 *
	3A	7.5% ^a^	17.7%	
	3B	18.5%	31.3%	
	4	40.5%	30.6%	
	5	25.0% ^b^	9.1%	
Age (yrs)		63 (50; 74)	66 (53; 75)	0.233
Sex: Male		55.3%	56.2%	0.849
Ethnicity	White	77.4%	79.0%	0.332
	Black	4.8%	2.9%	
	Asian	9.6%	8.7%	
	Chinese	1.9%	0.7%	
	Other	5.8%	6.2%	
	Unspecified	0.5%	2.5%	
Primary CKD Pathology	ADPKD	11.1%	6.5%	0.042 *^c^
	Diabetic Nephropathy	13.5%	8.7%	
	Glomerulonepgritis	18.3%	17.0%	
	Acute/Chronic TIN	2.9%	4.3%	
	Obstructive/Stones/Reflux	12.0%	9.8%	
	Renovascular/HTN/ Ischaemic	12.0%	12.7%	
	Vasculitis/SLE	6.7%	14.1%	
	Myeloma	2.4%	0.7%	
	Hereditary Nephropathy	1.4%	0.4%	
	Other	7.7%	8.7%	
	Uncertain Aetiology	12.0%	17.0%	
Cardiovascular Disease		20.7%	19.9%	0.840
Diabetes Mellitus		32.7%	25.4%	0.077
Davies’ Comorbidity Score		1 (1; 2)	1 (1; 2)	0.718
ACEi/A2RB	ACEi	33.4%	22.3%	0.560
	A2RB	21.5%	21.8%	0.742
	Both	2.4%	3.3%	0.201
Aldosterone Inhibitors		2.6%	2.9%	0.137
Baseline Serum Creatinine (umol/L)		222 (141; 322)	154 (119; 214)	<0.001 *
Baseline MDRD eGFR (mL/min/1.73 m^2^)		24 (15; 40)	34 (24; 48)	<0.001 *
uPCR mg/gCr		115 (24; 287)	25 (9; 73)	<0.001 *
uL-FABP ELISA (mcg/gCr)		7.8 (0; 31.1)	1.9 (0; 9.4)	<0.001 *
1 Year Serum Creatinine (umol/L)		278 (177; 438)	149 (109; 199)	<0.001 *
1 Year MDRD eGFR (mL/min/1.73 m^2^)		17 (10; 30)	37 (25; 52)	<0.001 *
Increase in Creatinine (%)		22.6 (13.3; 41.9)	−3.5 (−12.3; 1.5)	<0.001 *
Decrease in eGFR (mL/min/1.73 m^2^)		5 (3; 8)	−1 (−5; 1)	<0.001 *

CKD progression was defined as a decline in the MDRD eGFR by 5 mL/min or more, an increase in serum creatinine by 10% or more and renal death (initiation of renal replacement therapy). Group comparison was performed using Chi-square for categorical variables and Kruskal–Wallis test for continuous variables. Post hoc analysis for categorical variables was performed through observation of standardised residuals. * indicates statistical significance at *p* = 0.05, ^a^ indicates lower than expected frequency, ^b^ indicates higher than expected frequency and ^c^ indicates that observation of residuals did not identify a higher or lower than expected frequency for any of the categories. ACEi = angiotensin-converting enzyme inhibitors, A2RB = angiotensin 2 receptor blocker, CKD = chronic kidney disease, eGFR = estimate glomerular filtration rate, ELISA = enzyme-linked immunosorbent assay, HTN = hypertensive nephropathy, L = litre, m = metre, mcg = microgram, MDRD = Modification of Diet in Renal Disease, mg = milligram, mL = millilitre, mmol = millimol, SLE = systemic lupus erythematosus, TIN = tubulointerstitial nephritis, uL-FABP = urinary liver-type fatty acid binding protein, uPCR = urinary protein-to-creatinine ratio.

**Table 4:** Logistic regression models for the prediction of CKD progression by uL-FABP and uPCR during the first year of follow-up.

Outcome: CKD Progression, Predictor: uL-FABP				
	Unadjusted		+Age, Sex	
	OR (95% CI)	Sig	OR (95% CI)	Sig
uL-FABP	1.01 (1.00; 1.01)	0.002 *	1.01 (1.00; 1.01)	0.002 *
Outcome: CKD progression, Predictor: uPCR				
	Unadjusted		+Age, Sex	
	OR (95% CI)	Sig	OR (95% CI)	Sig
uPCR	1.00 (1.00; 1.01)	<0.001 *	1.00 (1.00; 1.01)	<0.001 *

Results are presented as Odds Ratios (OR) and coefficients with 95% confidence intervals (CIs). * denotes statistical significance (sig) at the level of *p* < 0.05. uL-FABP = urinary liver-type fatty acid binding protein, uPCR = urinary protein-to-creatinine ratio.

## Data Availability

Data cannot be made publicly available until the completion of all work relating to this study.

## Supplementary Materials

The following supporting information can be downloaded at: https://www.mdpi.com/article/10.3390/jpm13101481/s1, Table S1: Logistic regression models for the prediction of CKD progression by uLFABP and uPCR over the 2 years of follow-up; Table S2: Subgroup analysis of demographic and biochemical characteristics of participants’ with no proteinuria (uPCR < 50) and high uL-FABP levels (>8 mcg/gCr) that showed progression at year 1 of follow-up.
