# Supplementary material for: Urinary Liver-Type Fatty Acid Binding Protein, a Biomarker for Disease Progression, Dialysis and Overall Mortality in Chronic Kidney Disease
Source: J Pers Med. 2023 Oct 11;13(10):1481. doi: 10.3390/jpm13101481 (PMC10608048; doi:10.3390/jpm13101481)
Supplement: Supplementary file 1 [file jpm-13-01481-s001.zip › jpm-2641413-supplementary.pdf]

# Supplementary Material

## Table S1 : Logistic regression models for the prediction of CKD progression by uL-FABP and uPCR over the 2 years of follow-up

| Outcome: CKD progression, Predictor: uL-FABP | | | | |
|---|---|---|---|---|
| | Unadjusted | | + Age, Sex | |
| | OR (95% CI) | Sig | OR (95% CI) | Sig |
| **uL-FABP** | 1.01 (1.00; 1.01) | 0.015* | 1.01 (1.00; 1.01) | 0.016* |
| **Outcome: CKD progression, Predictor: uPCR** | | | | |
| | Unadjusted | | +Age, Sex | |
| | OR (95% CI) | Sig | OR (95% CI) | Sig |
| **uPCR** | 1.00 (1.00; 1.01) | <0.001* | 1.00 (1.00; 1.01) | <0.001* |

Results are presented as Odds Ratios (OR) and Co-efficient with 95% Confidence Intervals (CI). * denotes statistical significance (sig) at the level of p<0.05. uL-FABP= urinary liver type fatty-acid binding protein, uPCR= urinary protein to creatinine ratio.

## Table S2. Subgroup analysis of demographic and biochemical characteristics of participants' with no proteinuria (uPCR<50) and high uL-FABP levels (>8 mcg/gCr) that showed progression at year 1 of follow-up

| Demographics | | CKD progression | No CKD progression | p-value |
|---|---|---|---|---|
| **N** | | 12 | 23 | |
| **CKD stage** | 1-2 | - | 4.3% | 0.360 |
| | 3A | - | 13.0% | |
| | 3B | 16.7% | 17.4% | |
| | 4 | 75.0% | 43.5% | |
| | 5 | 8.3% | 21.7% | |
| **Age** | | 67 (SD 16) | 68 (SD 13) | 0.906 |
| **Sex: Male** | | 41.7% | 56.5% | 0.404 |
| **Ethnicity** | **White** | 75.0% | 78.3% | 0.218 |
| | **Black** | - | - | |
| | **Asian** | - | 13.0% | |
| | **Chinese** | - | - | |
| | **Other** | 25.0% | 8.7% | |
| | **Unspecified** | - | - | |
| **Primary CKD Pathology** | **APKD** | 33.3% | 13.0% | 0.535 |
| | **Diabetic Nephropathy** | 8.3% | 26.1% | |
| | **Glomerulonepgritis** | - | - | |
| | **Acute/Chronic TIN** | - | 13.0% | |
| | **Obstructive/Stones/Reflux** | - | - | |
| | **Renovascular/HTN/ Ischaemic** | 16.7% | 13.0% | |
| | **Vasculitis/SLE** | 8.3% | 8.7% | |
| | **Myeloma** | 8.3% | - | |
| | **Hereditary Nephropathy** | - | 4.3% | |
| | **Other** | 8.3% | 8.7% | |
| | **Uncertain Aetiology** | 16.7% | 13.0% | |
| **Cardiovascular Disease** | | 33.3% | 26.1% | 0.652 |
| **Diabetes Mellitus** | | 25.0% | 43.5% | 0.283 |
| **Davies' Comorbidity Score** | | 1.0 (0.25; 1.0) | 1.5 (1; 2) | 0.403 |
| **Baseline Serum Creatinine (umol/L)** | | 213 (SD 35) | 230 (SD 97) | 0.456 |
| **Baseline MDRD eGFR (ml/min/1.73m$^2$)** | | 23 (21; 27) | 21 (15; 39) | 0.889 |
| **uPCR mg/gCr** | | 32.1 (SD 10.9) | 25.5 (SD 14.2) | 0.169 |
| **uL-FABP ELISA (mcg/gCr)** | | 17.0 (13.0; 27.2) | 15.5 (12.1; 24.5) | 0.651 |
| **1 Year Serum Creatinine (umol/L)** | | 280 (SD 80) | 215 (SD 100) | 0.061 |
| **1 Year MDRD eGFR (ml/min/1.73m$^2$)** | | 19 (SD 8) | 29 (SD 15) | 0.026* |
| **Increase in Creatinine (%)** | | 23.1 (13.6; 36.4) | -6.8 (-17.2; 1.7) | <0.001* |
| **Decrease in eGFR (ml/min/1.73m$^2$)** | | 6 (3; 6) | -1 (-5; 1) | <0.001* |

CKD progression was defined as decline in the MDRD eGFR by 5ml/min or more, increase in serum creatinine by 10% or more and renal death (initiation of renal replacement therapy). Group comparison was performed using Chi-square for categorical variables and Kruskal Wallis test for continues variables. Post-hoc analysis for categorical variables was performed through observation of standardised residuals. * indicates statistical significance at p=0.05. APKD= adult polycystic kidney disease, CKD= chronic kidney disease, eGFR= estimate glomerular filtration rate, gCR= gram of creatinine, HTN= hypertensive nephropathy, L= litre, m= metre, mcg= microgram, MDRD= Modification of Diet in Renal Disease, mg= milligram, ml= millilitre, mmol= millimol, SLE= systemic lupus erythematosus, TIN= tubulointerstitial nephritis, uL-FABP= urinary liver type fatty-acid binding protein, uPCR= urinary protein to creatinine ratio
